# In vivo virus-free lineage tracing and live imaging reveal that NeuroD1 does not reprogram microglia into neurons

**DOI:** 10.7554/eLife.112083

**Published:** 2026-10-16

**Authors:** Xiaoyu Li, Yuxin Li, Yue Cao, Niewen Hu, Baozhi Yang, Pei Ouyang, Yuxiao Jin, Shuai Gao, Li Cao, Bo Peng, Yanxia Rao

**Affiliations:** 1 National Children’s Medical Center, Children’s Hospital, Institute for Translational Brain Research, State Key Laboratory of Medical Neurobiology, MOE Frontiers Center for Brain Science, Fudan SANS Neuroscience Center, Fudan University Shanghai China; 2 Department of Neurology, Zhongshan Hospital, Laboratory Animal Center, Fudan University Shanghai China; 3 Key Laboratory of Molecular Neurobiology, Ministry of Education, Navy Military Medical University Shanghai China; 4 Department of Neurosurgery, Huashan Hospital, Fudan University Shanghai China; 5 MOE Innovative Center for New Drug Development of Immune Inflammatory Diseases, Shanghai Key Laboratory of Gene Editing and Cell Therapy for Rare Diseases, Fudan University Shanghai China; Singapore Immunology Network Singapore; State University of New York Upstate Medical University United States

**Keywords:** NeuroD1, microglial reprogramming, lineage tracing, microglia-to-neuron conversion, Mouse

## Abstract

In situ glia-to-neuron conversion induced by a single transcription factor is a promising regenerative strategy for treating central nervous system injuries and neurodegenerative diseases. Previous studies reported that NeuroD1 can induce microglia-to-neuron cross-lineage conversion and improve recovery after brain injury. However, these findings remain controversial. To examine the ability of NeuroD1 in inducing microglia-to-neuron conversion, we employed virus-free genetic lineage tracing systems in mice to specifically induce NeuroD1 expression in microglia and track the cell fate of NeuroD1-expressing cells at multiple timepoints. Meanwhile, we longitudinally monitored NeuroD1-expressing microglia via two-photon imaging and characterized their transcriptional profile by single-cell RNA sequencing. All observations across these methods revealed that NeuroD1-expressing cells retained their microglia identity and cannot convert into neurons, under either physiological or injury conditions. Instead, sustained expression of NeuroD1 induced microglial apoptosis in vivo. Collectively, our findings provide strong evidence that NeuroD1 is not sufficient to induce microglia-to-neuron conversion. This study further highlights the importance of rigorous lineage tracing and cell fate mapping strategies for validating in situ glia-to-neuron conversion.

## Introduction

In the central nervous system, neurons have limited regenerative capacity; therefore, brain injury and neurodegeneration result in permanent neuronal loss. In contrast, glial cells, especially microglia, exhibit a high regenerative capacity (Varvel et al., 2012; Huang et al., 2018; Li et al., 2023). In situ reprogramming glial cells into neurons offers a promising strategy for neuronal regeneration and functional recovery in brain injuries and neurodegenerative disease. Recently, single genes such as *Neurod1* (Guo et al., 2014), *Pax6* (Heins et al., 2002), *Sox2* (Tai et al., 2021; Niu et al., 2013; Niu et al., 2015), *Ascl1* (Chanda et al., 2014), and *Ptbp1* (Qian et al., 2020; Zhou et al., 2020) have been reported to induce glia-to-neuron cell fate conversions within the neuroectoderm-origin cells, transforming astrocytes, oligodendrocyte precursor cells, or Muller glia into neurons, shedding light on neurodegenerative therapies.

However, before clinical translation, these approaches require rigorous validation using stringent and robust approaches. Unbiased and well-controlled lineage tracing and unambiguous live imaging are regarded as gold standards for validating glia-to-neuron conversion (Svendsen and Sofroniew, 2022; Rao et al., 2021). Indeed, several studies employing rigorous lineage tracing and live imaging techniques have failed to observe some intra-lineage glia-to-neuron conversions with single-gene manipulation (Hoang et al., 2023; Yang et al., 2023; Xie et al., 2022; Guo et al., 2022; Hoang et al., 2022; Chen et al., 2022; Xie et al., 2023; Leib et al., 2022; Wang et al., 2021), including NeuroD1 (Xie et al., 2023; Leib et al., 2022; Wang et al., 2021). In addition to intra-lineage conversion, Nakashima et al. have reported that NeuroD1 can induce microglia-to-neuron cross-lineage conversion (Matsuda et al., 2019). We previously conducted lineage tracing using a virus-based tracing system, in vitro live-cell imaging, and microglia ablation, and demonstrated that the reported cross-lineage conversion of microglia into neurons may be attributed to experimental artifacts, for example, off-target transduction from the virus (Rao et al., 2021). Although Nakashima et al. argued that the phenomenon we observed was due to insufficient *Neurod1* expression in microglia (Matsuda and Nakashima, 2021), our comparison of the data from both studies (Rao et al., 2021; Matsuda et al., 2019) indicates that our *Neurod1* expression levels are comparable to, or even higher than, those reported in their study (Rao and Peng, 2022). Besides, Nakashima et al. reported that *Neurod1* could induce the microglia-to-neuron conversion at brain injury sites in vivo and promote recovery after brain injury (Irie et al., 2023). Such conflicting reports are of particular concern because they directly influence whether glia-to-neuron reprogramming could be translated into clinical. To settle this controversy, we employed a virus-free genetic lineage tracing system to avoid potential experimental artifacts, longitudinal two-photon live imaging to directly monitor the changes in NeuroD1-expressing microglia in vivo, and single-cell RNA sequencing (scRNA-seq) to characterize their transcriptional profiles. Our findings provide strong evidence that microglia do not undergo conversion into neurons in vivo, under either physiological or pathological conditions. Our study highlighted the importance of employing rigorous lineage tracing, longitudinal live-cell imaging, and multimodal validation in evaluating glia-to-neuron reprogramming approaches.

## Results

### Genetic lineage tracing reveals that NeuroD1 expression in microglia fails to induce neuron conversion in vivo

We previously reported that ectopic expression of NeuroD1 using a lentiviral vector does not induce the microglia-to-neuron conversion, but triggers microglial cell death (Rao et al., 2021). However, most viral tools designed for microglia exhibit limited efficiency and specificity in the brain*,* but display off-target transduction to neurons and astrocytes (Rao et al., 2021; Xie et al., 2023; Rosario et al., 2016). This limitation was observed in either lentivirus or adeno-associated virus (AAV) (with different serotypes 6, 8, and 9), and irrespective of the use of myeloid *Cx3cr1* or *Cd68* promoters (Figure 1—figure supplement 1). Similar observations have been reported in our previous study and other groups (Rao et al., 2021; Xie et al., 2023; Rosario et al., 2016).

**Figure 1.**
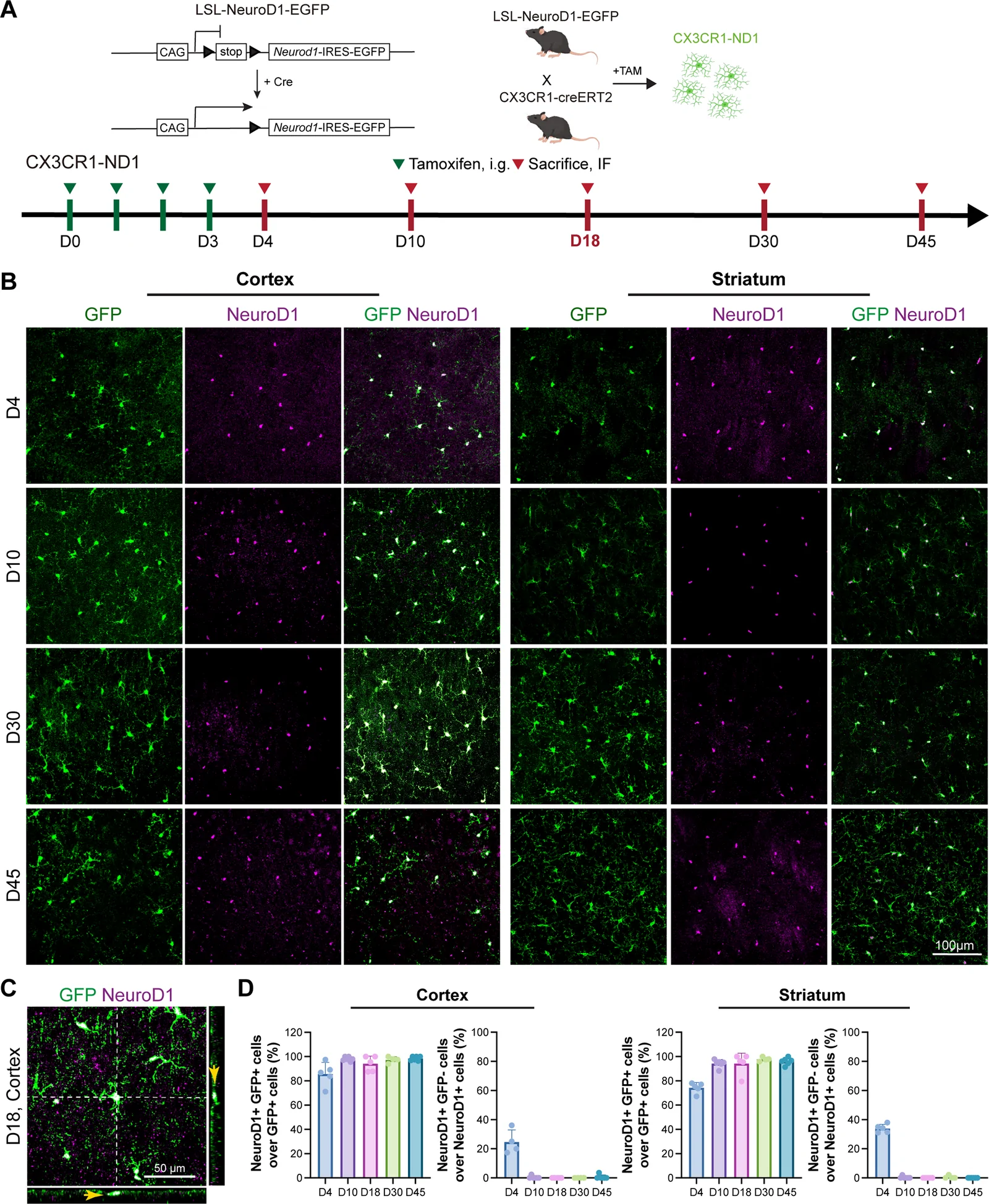
Virus-free genetic induction can successfully induce NeuroD1 expression in microglia. (**A**) Schematic of the specific expression of NeuroD1, lineage tracing of microglia, and experimental timeline. D, day; i.g., intragastric gavage. (**B**) Representative confocal images at D4, D10, D30, and D45 showing that NeuroD1 is robustly co-expressed in GFP-positive cells after Cre recombination. (**C**) Representative confocal images at D18 showing that NeuroD1 is robustly co-expressed in GFP-positive cells after Cre recombination. (**D**) Quantification of the percentage of NeuroD1^+^GFP^+^ cells among GFP^+^ cells and NeuroD1^+^GFP^-^ cells among NeuroD1^+^, revealing NeuroD1 is highly co-expressed with GFP. N=4–6 mice for each group. Data are presented as mean ± SD.

Direct evidence for glia-to-neuron conversion by NeuroD1 via a virus-free expression system, particularly genetic lineage tracing, is lacking. Genetic lineage tracing is essential for avoiding experimental artifacts from viruses (Svendsen and Sofroniew, 2022; Rao et al., 2021), including viral leakage and/or Cre-independent recombination. Thus, we utilized transgenic animals to specifically induce NeuroD1 expression in microglia and track the cell fate of NeuroD1-expressing microglia. We therefore crossed macrophage/microglia-targeting CX3CR1-CreER mouse line or a microglia-specific TMEM119-CreER mouse line (Bedolla et al., 2024; Yona et al., 2013; Kaiser and Feng, 2019), with an inducible NeuroD1-expressing mouse line Rosa26-LoxP-Stop-LoxP-NeuroD1-IRES-GFP (LSL-NeuroD1-GFP) (Li et al., 2019)*,* to obtain CX3CR1-CreER::LSL-NeuroD1-GFP mice (hereafter, CX3CR1-ND1) and TMEM119-CreER::LSL-NeuroD1-GFP mice (hereafter, TMEM119-ND1 for short). Meanwhile, CX3CR1-CreER::Ai14 (CX3CR1-Ai14) and TMEM119-CreER::Ai14 (TMEM119-Ai14) mice were used as controls (Figure 1A, Figure 1—figure supplement 2). Following tamoxifen treatment, the vast majority of GFP-positive cells were continuously co-expressed with NeuroD1 (Figure 1B), enabling us to track NeuroD1-expressing cells for lineage tracing. Meanwhile, we observed that these GFP-positive cells retained the characteristic morphology of microglia, exhibiting typical highly ramified processes and small cell bodies (Figure 1, Figure 1—figure supplement 2).

**Figure 1—figure supplement 1.**
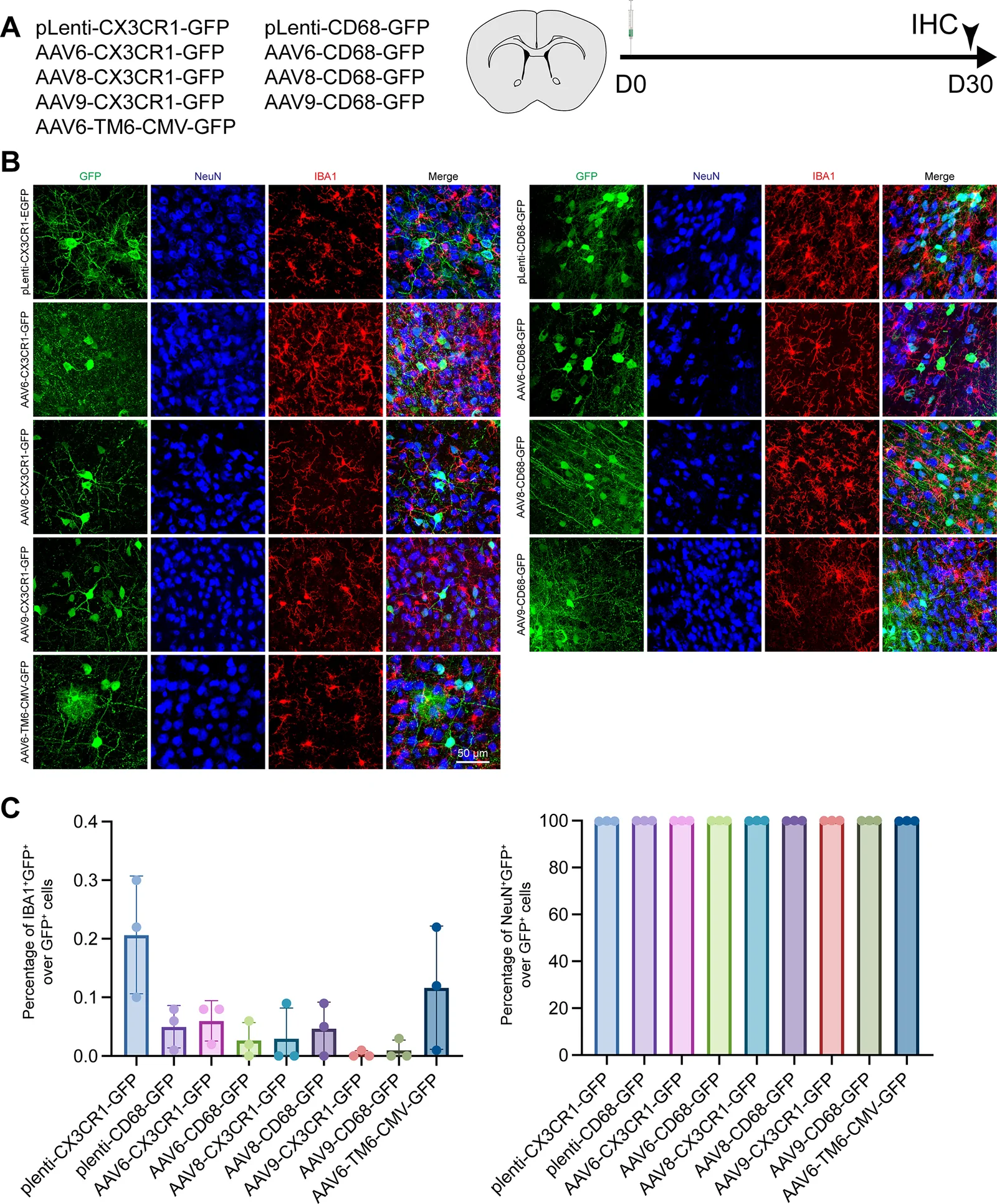
Lentivirus- or AAV-based infection caused leaky neuronal expression in vivo. (**A**) The study design shows the different viruses used here. (**B**) Representative confocal images showing the expression of GFP, IBA1, and NEUN in the brains of the lentivirus- or AAV-infected mice. (**C**) The efficacy of infection was quantified, and the data are presented as the ratio of IBA1^+^GFP^+^/GFP^+^ cells. N = 3 mice for each group. Data are presented as mean ± SD. Each data point represents one mouse.

**Figure 1—figure supplement 2.**
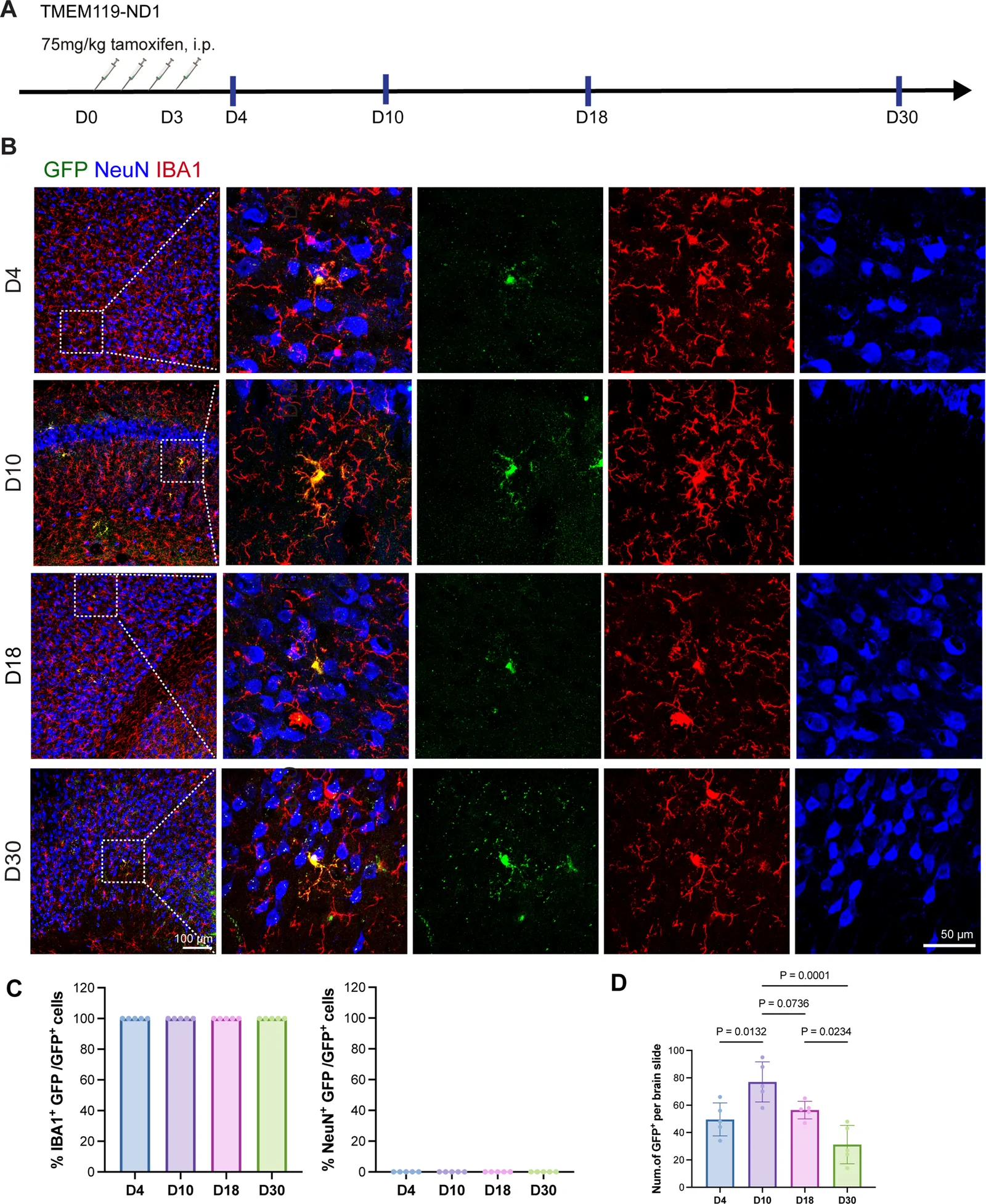
NeuroD1-expressing microglia are co-labeled with the microglial marker IBA1 but not the neuronal marker NeuN. (**A**) Experimental timeline. (**B**) Representative confocal images at D4, D10, D18, and D30 showing that GFP is robustly co-expressed in IBA1^+^ cells but not NeuN^+^ cells after Cre recombination. (**C**) Quantification of IBA1^+^GFP^+^ double-positive cells in GFP^+^ cells and NeuN^+^GFP^+^ in GFP^+^ cells at D4, D10, D18, and D30. N = 5 mice for each group. (**D**) Quantification of GFP^+^ cells per brain slide in TMEM119-ND1 mice. N = 5 mice for each group. Data are presented as mean ± SD. Each data point represents one mouse.

**Figure 2.**
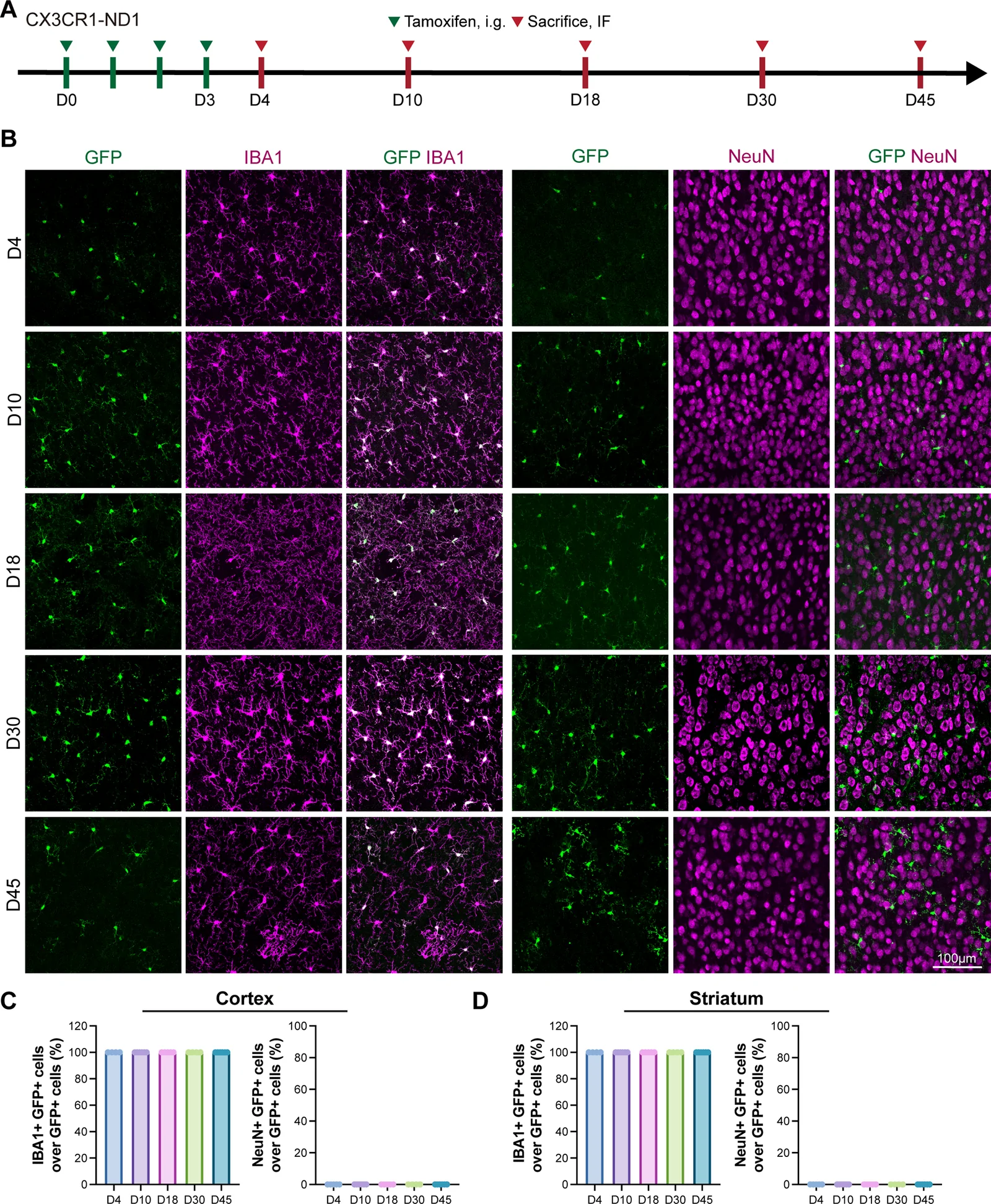
Sustained NeuroD1 expression in microglia does not induce microglia-to-neuron conversion across time. (**A**) Experimental timeline. D, day; i.g., intragastric gavage. (**B**) Representative confocal images at D4, D10, D18, D30, and D45 showing that GFP is co-labeled with IBA1 but not co-labeled with NeuN in the cortex. (**C**) Quantification of IBA1+GFP+ double-positive cells among GFP+ cells or NeuN+GFP+ double-positive cells among NeuN+ in the cortex at D4, D10, D18, D30, and D45. N = 4 or 5 mice for each group. (**D**) Quantification of IBA1+GFP+ double-positive cells among GFP+ cells or NeuN+GFP+ double-positive cells among NeuN+ in the striatum at D4, D10, D18, D30, and D45. N = 4 or 5 mice for each group. Data are presented as mean ± SD. Each data point represents one mouse.

We subsequently examined the fate of GFP-positive cells at multiple timepoints, at 4, 10, 18, 30, and 45 days post-tamoxifen induction. Immunostaining for microglia and neuron markers revealed that all GFP-positive cells were consistently co-localized with the microglial marker IBA1, but they did not express neuronal markers, NeuN, TUJ1, or MAP2 within CX3CR1-ND1 mouse brain (Figure 2, Figure 2—figure supplement 1A–D). The observations were the same in TMEM119-ND1 (Figure 1—figure supplement 2). These findings indicate that, despite sustained NeuroD1 ectopic expression by genetic modification, microglia retain their microglial identity but do not acquire a neuronal cell fate.

**Figure 2—figure supplement 1.**
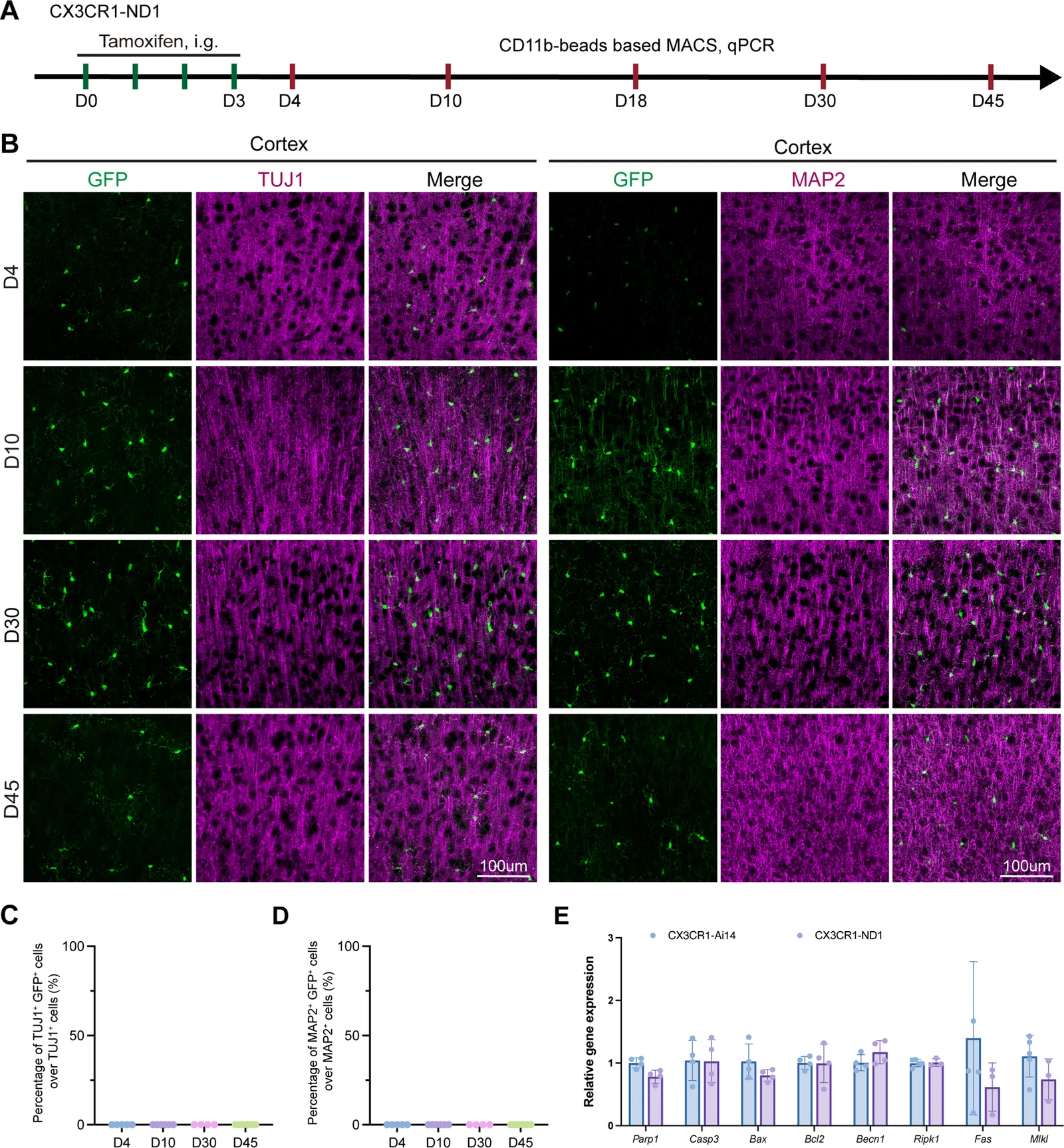
Ectopic NeuroD1 expression in microglia does not induce microglia-to-neuron conversion, as GFP does not co-localize with TUJ1 and MAP2. (**A**) Experimental timeline. (**B**) Representative confocal images at D4, D10, D30, and D45 showing that GFP is not co-labeled with TUJ1 or MAP2 in the brain. (**C**) Quantification of TUJ1^+^GFP^+^ double-positive cells among TUJ1^+^ cells at D4, D10, D30, and D45. N = 4 or 5 mice for each group. (**D**) Quantification of MAP2^+^GFP^+^ double-positive cells among MAP2^+^ cells at D4, D10, D30, and D45. N = 4 or 5 mice for each group. (**E**) Relative gene expression level at D18. N = 4 mice for each group. Data are presented as mean ± SD. Each data point represents one mouse.

### Ectopic NeuroD1 expression induces microglial apoptosis rather than neuronal conversion

**Figure 3.**
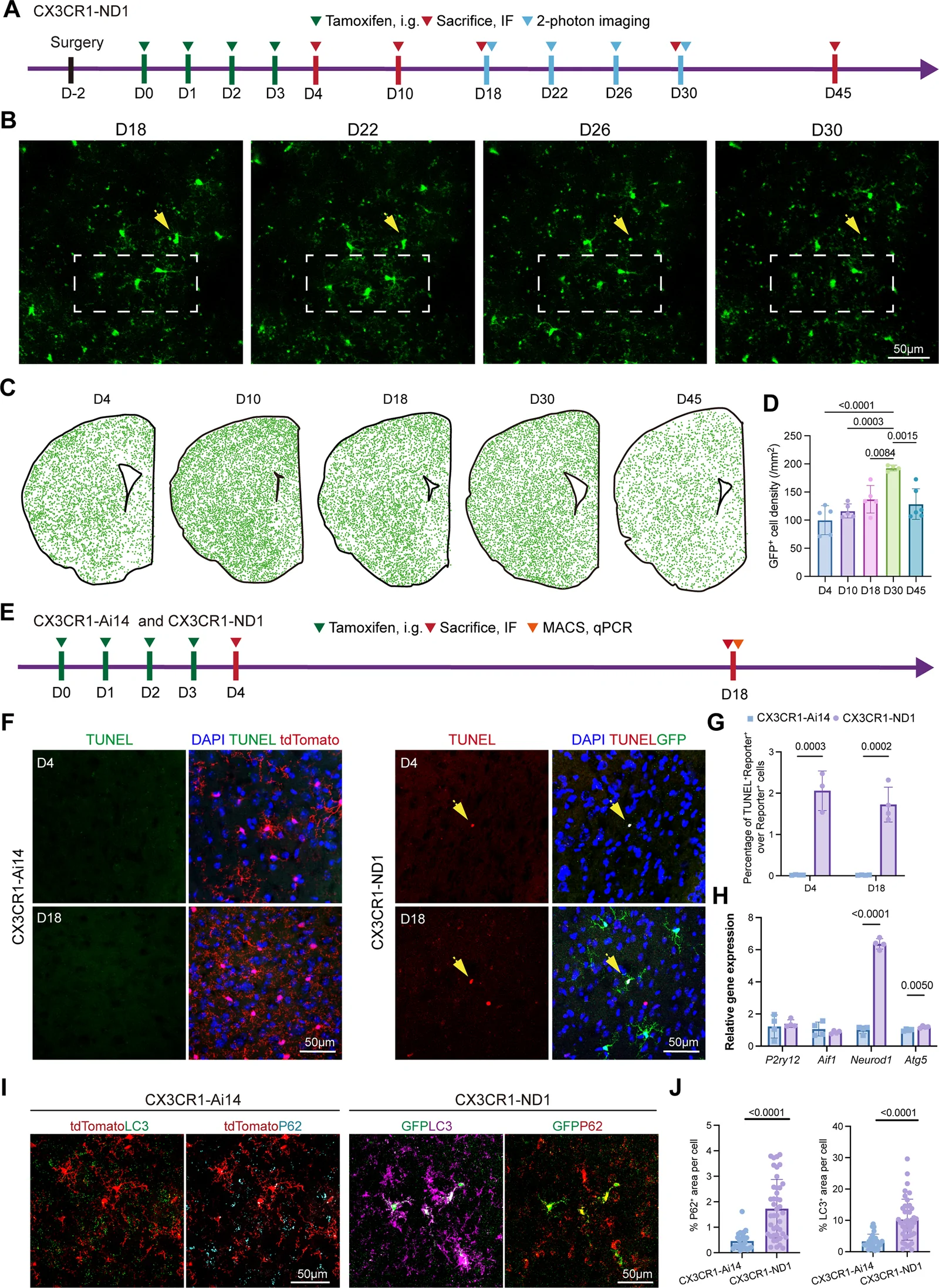
NeuroD1-expressing microglia retain their microglial characteristic morphology, but undergo progressive cell loss over time. (**A**) Experimental timeline. D, day; i.g. intragastric gavage. (B) Representative images showing GFP-positive cells with highly ramified morphologies characteristic of microglia rather than neurons. The white rectangle indicates no morphological change, the yellow arrow indicating the GFP+ cell loss over time. (**C**) Representative images illustrating changes in the number of GFP-positive cells over time (green dots indicate GFP-positive cells). (**D**) Quantification of GFP-positive cell density. N = 4–6 mice per timepoint. Data are presented as mean ± SD. One-way ANOVA with Tukey’s multiple-comparison test. (**E**) Experimental timeline. (**F**) Representative confocal image shows TUNEL+ Reporter+ cells are increased in CX3CR1-ND1 animals. (**G**) Quantification of TUNEL+ Reporter+ cells at D4 and D18, unpaired *t*-test. Data are presented as mean ± SD. (**H**) Relative gene expression level at D18, unpaired *t*-test. Data are presented as mean ± SD. (**I**) Representative confocal image shows the LC3 and P62 levels in CX3CR1-Ai14 and CX3CR1-ND1 mice. Scale bar, 50 μm. (**J**) Quantification of P62+ and LC3+ area per cell. Unpaired *t*-test. N = 3~ 6 per group. Data are presented as mean ± SD. Each data point represents one mouse, except in (J), where each data point represents one cell.

Given the possibility that genetically labeled cells may lose or downregulate the reporter, such as GFP expression during cell fate conversion (Guo et al., 2024), we further monitored the cell fate of GFP-labeled microglia in vivo using two-photon microscopy (Figure 3A). We repeatedly imaged GFP-positive cells in the sensorimotor cortex at 4-day intervals, beginning on day 18, and continuing throughout day 30 post-tamoxifen administration (Figure 3A). This strategy allowed us to directly track individual NeuroD1-expressing microglia over time. The longitudinal live-cell imaging revealed that there was no obvious morphological transition during the imaging period (Figure 3B, white rectangle, and Figure 3—video 1). Instead, the GFP-positive cells consistently exhibited a highly ramified morphology with short and motile processes, which were typical morphological features of microglia rather than neurons (Figure 3B, white rectangle, and Figure 3—video 1). Meanwhile, we observed that some GFP-labeled microglia progressively disappeared during the imaging period (Figure 3B, yellow arrow, and Figure 3—video 1). These observations indicate sustained ectopic NeuroD1 expression could not induce microglia-to-neuron conversion, but induce microglial cell loss in vivo. These in vivo time-lapse imaging observations were consistent with our previous finding from in vitro live-cell imaging (Rao et al., 2021). We further quantified numbers of GFP-labeled microglia at multiple timepoints after tamoxifen induction. The number of GFP-labeled cells initially increased and reached a peak at day 30 post-tamoxifen induction, but subsequently declined by day 45 (Figure 3C and D). The progressive loss of GFP-labeled microglia was also observed in TMEM119-ND1 brain (Figure 1—figure supplement 2D). These findings further supported that NeuroD1 expression in microglia induces progressive cell loss over time in vivo.

**Figure 3—video 1.** This video shows that GFP^+^ cells did not convert to neurons in vivo via two-photon imaging at 18, 22, 26, and 30 days post-tamoxifen administration. Refers to Figure 3.

To investigate the mechanisms underlying NeuroD1-expressing microglia loss, we first asked whether the progressive loss of NeuroD1-expressing microglia was due to cell apoptosis. To this end, we assessed apoptosis by TUNEL staining in CX3CR1-ND1 and CX3CR1-Ai14 mice (Figure 3E). In line with the gradual disappearance of GFP-positive cells, we observed that there was hundred times higher proportion of TUNEL-positive cells among reporter-positive cells in CX3CR1-ND1 mice than that of controls, increasing from approximately 0.01% in controls to 1.7% (at D18) in NeuroD1-expressing cells (Figure 3F and G). This indicates that NeuroD1 expression accelerates microglia apoptosis in vivo. We next asked about the molecular mechanisms through which NeuroD1 induces microglia cell death. Microglia were isolated from CX3CR1-ND1 and CX3CR1-Ai14 brains using CD11b-based magnetic separation at day 18 post-tamoxifen induction (Figure 3E, Figure 2—figure supplement 1A). We then performed qPCR to examine the expression of genes involved in apoptosis (*Parp1*, *Casp3*, *Bax*, *Bcl2*), necrosis (*Ripk1*, *Fas*, *Mlkl*), and autophagy (*Becn1* and *Atg5*) (Figure 2—figure supplement 1E and Figure 3H; Rao and Peng, 2022; Noguchi et al., 2020; Chen et al., 2018). Among the genes examined, *Atg5*, a key regulator of autophagy (Changotra et al., 2022), was significantly upregulated in cells isolated from CX3CR1-ND1 compared with controls (Figure 3H). We therefore examined autophagic markers following NeuroD1 induction. In CX3CR1-ND1 mice, reporter^+^ cells exhibited about fourfold higher LC3 and P62 levels than controls, as shown by immunostaining (Figure 3I and J), suggesting accumulation of autophagosomes markers and impaired autophagic flux. Taken together, these data indicate that sustained ectopic NeuroD1 expression induces microglia apoptosis, accompanied with autophagic dysregulation, leading to NeuroD1-expressing microglia loss in vivo, rather than microglia-to-neuron conversion.

### NeuroD1 fails to reprogram microglia into neurons even in the injured brain

Previous studies suggest that injury environments might precondition glial cells for fate reprogramming. For example, Heinrich et al. demonstrated that a stab wound prior to virus delivery is necessary to stimulate NG2 glia to neuron conversion by *Sox2* (Heinrich et al., 2014). Similarly, Irie et al. showed that *Neurod1* can directly reprogram microglia/macrophages into neurons at the lesion site following transient middle cerebral artery occlusion (Irie et al., 2023). These findings raise the possibility that the failure of NeuroD1-mediated microglia-to-neuron conversion under physiological conditions may be attributed to the absence of injury-induced reprogramming permissive microenvironment.

**Figure 4.**
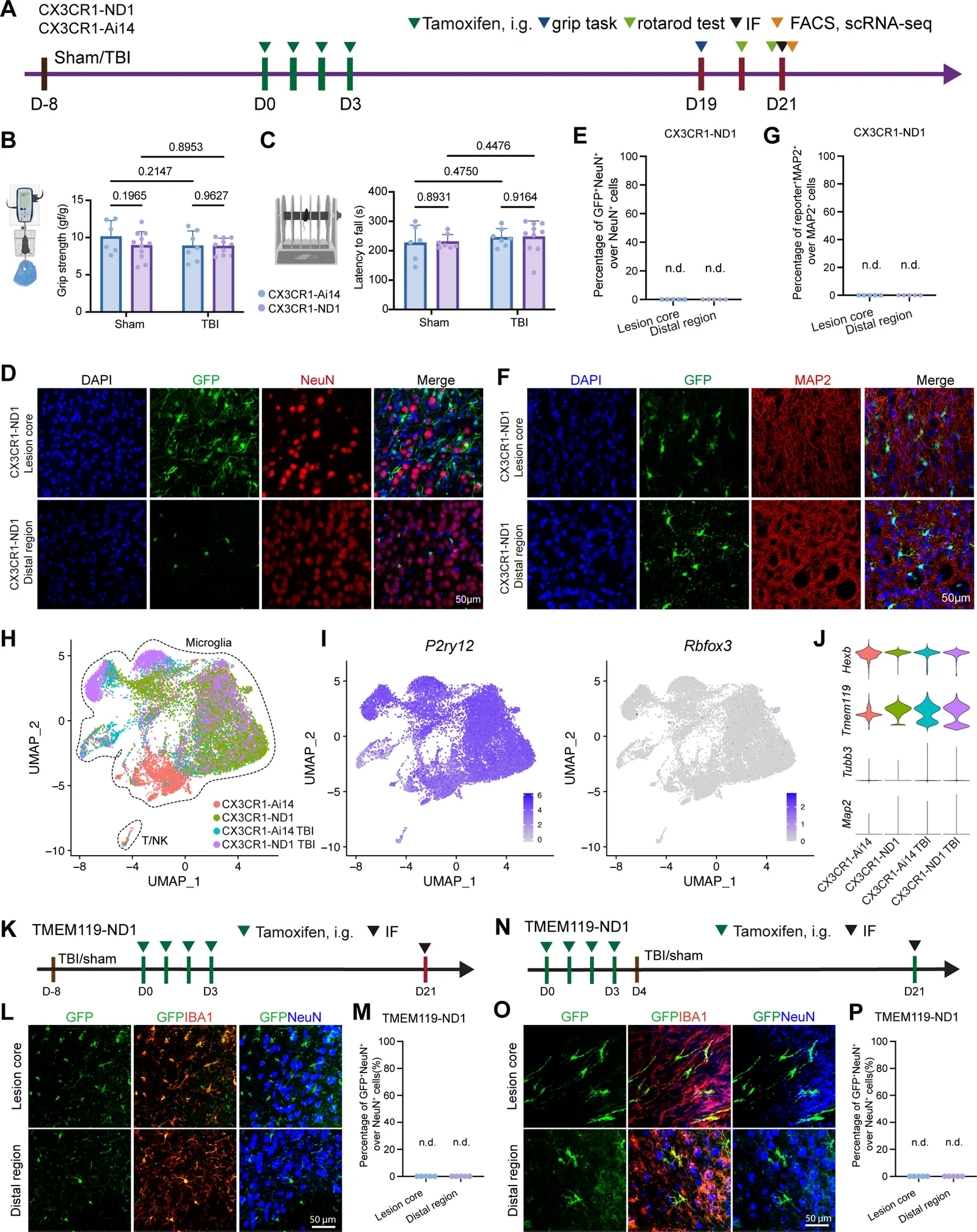
NeuroD1 does not induce microglia-to-neuron conversion even in an injury conditioned microenvironment. (**A**) Experimental design. D, day; i.g., intragastric gavage. (**B**) Quantification of grip strength showing no improvement in NeuroD1-expressing TBI mice. Two-way ANOVA with multiple comparisons. (**C**) Quantification of rotarod performance showing no improvement in NeuroD1-expressing TBI mice. Two-way ANOVA with multiple comparisons. (**D**) Representative confocal images of DAPI, GFP, and NeuN co-staining results in CX3CR1-ND1 TBI mice, showing no co-localization of GFP with NeuN in either the lesion core or distal regions. Scale bar, 50 μm. (**E**) Quantification of the percentage of GFP+NeuN+ cells among total NeuN+ cells in both the lesion area and distal area. n.d., not detected. (**F**) Representative confocal images of DAPI, GFP, and MAP2 in CX3CR1-ND1 mice, showing no co-localization of GFP with MAP2 in either the lesion core or distal regions. Scale bar, 50 μm. (**G**) Quantification of the percentage of GFP+MAP2+ cells among total MAP2+ cells in the lesion area and distal region. n.d., not detected. (**H**) UMAP reduction shows the cell types of the FACS-sorted reporter+ cells in each group; note that there are no neurons. (**I**) Feature plot showing that the microglia-specific marker P2ry12 was highly expressed even under TBI conditions, but the neuron-specific marker Rbfox3 was barely expressed. (**J**) Violin plot showing the expression of microglia-specific genes (Hexb and Tmem119) and neuron-specific genes (Tubb3 and Map2) in four groups. (**K**) Experiment timeline of TMEM119-ND1 mice, TBI was performed before tamoxifen induction. (**L**) Representative confocal images of GFP, IBA1, and NeuN in TMEM119-ND1 mice, showing no co-localization of GFP with NeuN in either the lesion core or distal regions. Scale bar, 50 μm. (**M**) Quantification of the percentage of GFP+NeuN+ cells among total NeuN+ cells in the lesion area and distal region. n.d., not detected. (**N**) Experiment timeline of TMEM119-ND1 mice; TBI was performed after tamoxifen induction. (**O**) Representative confocal images of GFP, IBA1, and NeuN in TMEM119-ND1 mice, showing no co-localization of GFP with NeuN in either the lesion core or distal regions. Scale bar, 50 μm. (**P**) Quantification of the percentage of GFP+NeuN+ cells among total NeuN+ cells in the lesion area and distal region. n.d., not detected. For behavioral experiments (B, C), N = 6–10 mice per group. For histological quantification (E, G, M, P), N = 5 mice per group. Data are presented as mean ± SD. Each data point represents one mouse. Statistical significance in panels B and C was assessed using two-way ANOVA followed by multiple-comparison tests.

To test this possibility, we established a moderate traumatic brain injury (TBI) in the motor cortex in CX3CR1-ND1 mice as previously described (Cai et al., 2025), followed by tamoxifen induction to activate NeuroD1 expression in microglia (Figure 4A). If the injury environment stimulates NeuroD1-mediated microglia-to-neuron conversion, the newborn neurons would contribute to motor function recovery after TBI. We therefore performed a series of behavior tests to assess motor recovery at 19 days post-tamoxifen induction (Figure 4A). In grip strength task, the CX3CR1-ND1 mice showed comparable performance to that of controls, with no significant differences in the strength between groups (Figure 4B). Similarly, no improvements were observed in the rotarod task, as reflected by a comparable latency to fall between groups (Figure 4C). These results indicate that NeuroD1 expression in microglia does not improve the motor function recovery in the TBI model. We next directly examined the cell fate of NeuroD1-expressing microglia in the injured brain. Immunostaining analysis showed that GFP-labeled NeuroD1-expressing microglia did not co-localize with the neuronal marker NeuN (Figure 4D and E) or MAP2 (Figure 4F and G), both in the lesion core and distal regions. To further figure out the cell fate of microglia after NeuroD1 induction, we conducted scRNA-seq to characterize the transcriptional profile of NeuroD1-expressing cells (Figure 4A). Both tdTomato and GFP-positive cells were isolated from CX3CR1-Ai14 and CX3CR1-ND1 brains respectively, using flow cytometry (Figure 4—figure supplement 1A), under both physiological and TBI conditions. These cells were subsequently subjected to scRNA-seq (Figure 4—figure supplement 1B–E). In line with the immunofluorescent findings, when we annotated sorted reporter-positive cells, we did not identify any neuronal cluster, even under injury conditions (Figure 4H, Figure 4—figure supplement 1E). Additionally, the reporter-positive cells still strongly expressed microglia markers, including *P2ry12*, *Hexb*, and *Tmem119* (Figure 4I and J), but did not express neuronal markers, such as *Rbfox3*, *Tubb3*, and *Map2* (Figure 4I and J). Collectively, these findings demonstrate that NeuroD1 fails to induce microglia-to-neuron conversion, even under an injury condition.

**Figure 4—figure supplement 1.**
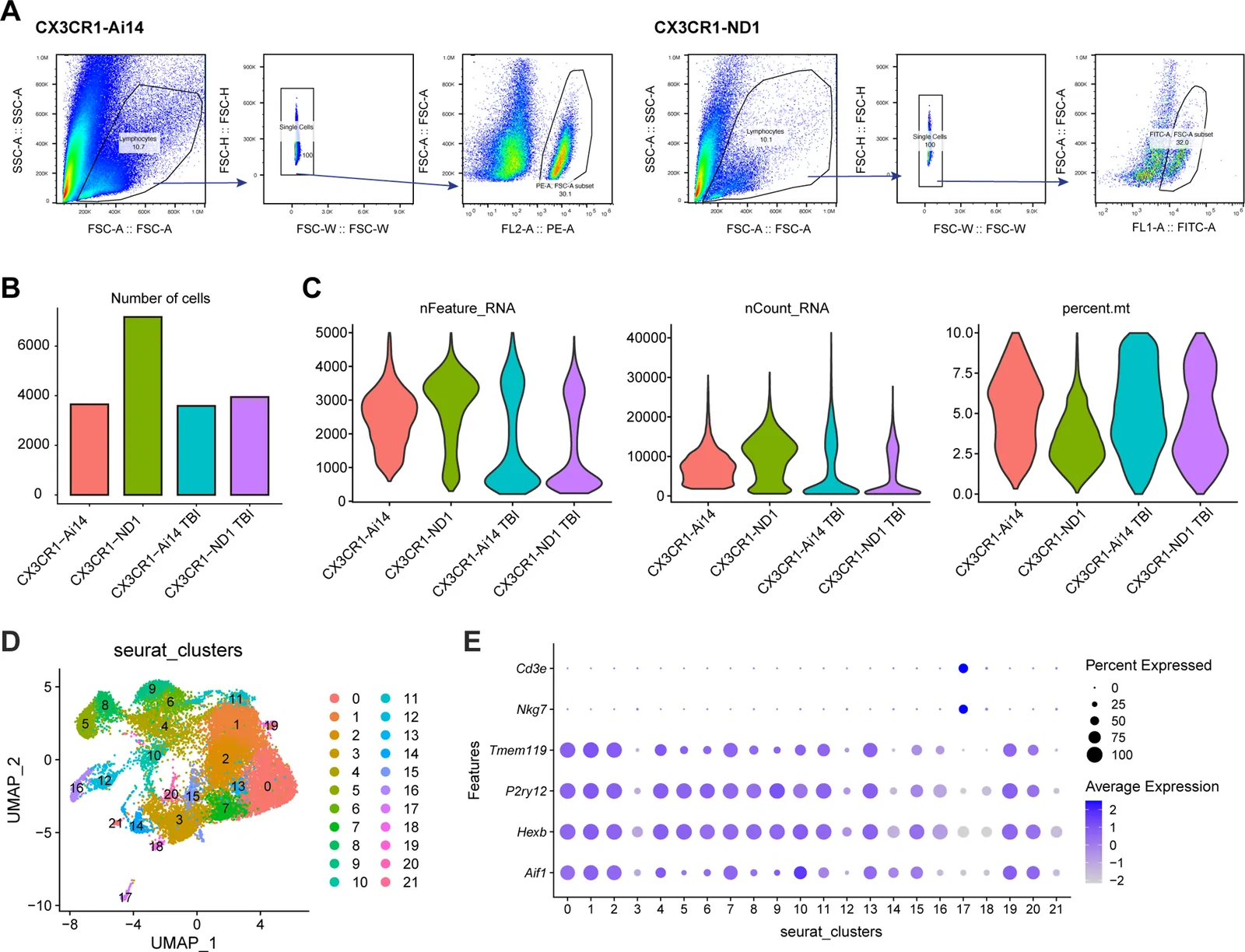
FACS gating strategy and supplemental quality control information of scRNA-seq data. (A) Gating strategy of CX3CR1-Ai14 and CX3CR1-ND1 mice. (B) Number of cells (after quality control) per group. (C) Violin plot shows the number of genes detected in each cell (nFeature_RNA), total number of molecules detected within a cell (nCount_RNA), and the distribution of mitochondrial genes in each cell (percent.mt) among the four groups. (D) UMAP plot shows all cells were divided into 22 unsupervised clusters with resolution = 1. (E) Dotplot shows microglia, T cell, and NK cell-specific markers' expression over 22 clusters.

Given that tamoxifen was administered after TBI induction in CX3CR1-ND1 mice, when circulating monocytes and macrophages were recruited into the injury region, the CX3CR1-CreER system can also label these peripheral myeloid cells during this period (Yona et al., 2013; Cai et al., 2025). To exclude the possibility that recruited myeloid cells misled the lineage tracing result, we repeated the same experiments in TMEM119-ND1 mice (Figure 4K). The TMEM119-CreER system allows us to restrict NeuroD1 and GFP expression specifically to resident microglia in the brain (Bedolla et al., 2024). Consistent with the observations in CX3CR1-ND1 mice, we did not detect any GFP and NeuN co-localized cells in either the lesion core or regions distal to the injury site (Figure 4L). In addition, we reversed the sequence by conducting TBI after tamoxifen treatment, in turn labeling resident microglia before the recruitment of peripheral myeloid cells (Figure 4N). Similarly, we observed no GFP-positive cells co-expressing the pan neuron marker NeuN in this condition as well (Figure 4O). These findings further proved that NeuroD1 does not reprogram resident microglia into neurons even under injury environments.

## Discussion

In the current study, we employed a virus-free genetic lineage tracing system based on CX3CR1-CreER and TMEM119-CreER to trace the cell fate of NeuroD1-expressing cells, instead of viral-based approaches. Through immunostaining assays at multiple timepoints, two-photon live-cell imaging, and scRNA sequencing, we systematically assessed the cell fate of NeuroD1-expressing microglia under both physiological and injury conditions. Collectively, all concordant observations provide strong evidence that NeuroD1-expressing microglia consistently retained their microglial identity and failed to acquire neuronal cell fate. These findings in the current study contrast with previous reports claiming NeuroD1-mediated microglia-to-neuron conversion (Matsuda and Nakashima, 2021; Irie et al., 2023) and instead support the notion that NeuroD1 is insufficient to induce microglia-to-neuron conversion. We speculated that the observed microglia-to-neuron conversion reported in previous studies may have resulted from off-target viral expression, which leads to the misinterpretation of the cell source of the observed neurons (Rao et al., 2021; Leib et al., 2022; Wang et al., 2021; Rao and Peng, 2022).

Previous studies have highlighted the controversial observations in validating direct glia-to-neuron reprogramming (Rao et al., 2021; Rao and Peng, 2022). While several studies demonstrated that the single transcription factors, such as SOX2, ASCL1, or NeuroD1, could convert glial cells into neurons, subsequent investigations using rigorous lineage tracing or live imaging have failed to reproduce some of these intra-lineage conversions (Xie et al., 2023). In fact, our longitudinal live-cell imaging revealed a gradual loss of NeuroD1-expressing microglia over time. These findings were consistent with our previous results that ectopic NeuroD1 induced microglia cell apoptosis (Rao et al., 2021). The gradual disappearance of NeuroD1-expressing microglia over time, LC3/P62 accumulation, and increased TUNEL-positive microglia collectively indicate that NeuroD1 compromises microglial homeostasis and facilitates cell death rather than inducing microglia-to-neuron conversion. Although the present study suggests that in situ microglia-to-neuron conversion remains technically challenging and requires novel approaches, our team has developed an alternative therapeutic strategy, that is, microglia replacement by bone marrow transplantation (Mr BMT) (Rao et al., 2025; Xu et al., 2020). Mr BMT allows healthy microglia carrying normal or functional genes to replace diseased microglia carrying mutated genes, thereby providing an avenue to cure diseases associated with primary microgliopathies (Rao et al., 2025). Indeed, a simplified version of Mr BMT has been successfully translated into clinical practice and was shown to halt disease progression in patients with adult-onset leukoencephalopathy with axonal spheroids and pigmented glia, supporting Mr BMT as a promising cell-based therapy in neurological diseases (Wu et al., 2025).

Together, these findings underscore the importance of stringent lineage tracing approaches and multimodal validation strategies when evaluating cell fate conversion. Although glia-to-neuron reprogramming remains a promising avenue for regenerative medicine, accumulating studies, including the present study, raise caution in interpreting in vivo glia-to-neuron conversion without rigorous validation of cellular origin and cell fate transition, particularly when considering the clinical translation of such approaches.

## Materials and methods

### Animals

CX3CR1-CreER [B6.129P2(C)-*Cx3cr1*^tm2.1(cre/ERT2)Jung^/J, Stock No: 020940], Ai14 [B6.Cg-Gt(ROSA)26Sor^tm14(CAG-tdTomato)Hze^/J, Stock No: 007914], TMEM119-CreER (C57BL/6-*Tmem119*^em1(cre/ERT2)Gfng/J^, Stock No: 031820), and LSL-NeuroD1-GFP [Gt(ROSA)26Sor^tm1(Neurod1-EGFP)Able^/BfriJ, Stock No: 034697] mice were purchased from The Jackson Laboratory. CX3CR1-CreER mice were crossed with LSL-NeuroD1-EGFP mice to obtain CX3CR1-CreER^+/CreER^::LSL-NeuroD1-EGFP^+/+^ (CX3CR1-ND1) mice. Similarly, CX3CR1-CreER mice were crossed with Ai14 mice to obtain CX3CR1-CreER::Ai14 (CX3CR1-Ai14) mice. All animals were housed in the Animal Facility of the Department of Laboratory Animal Science at Fudan University on a 12 h light‒dark cycle with food and water provided ad libitum. No significant sex differences were observed in this study. All animal experiments were conducted in accordance with institutional guidelines for the care and use of laboratory animals and were approved by the Ethics Committee of Fudan University (approval no. 202106045S). All efforts were made to minimize animal suffering and the number of animals used.

### Drug administration

For mice aged 8 weeks or older, CX3CR1-ND1, CX3CR1-Ai14, TMEM119-ND1, and TMEM119-Ai14 mice, tamoxifen at 150 mg/kg body weight was administered via intragastric gavage (i.g.) or intraperitoneal injection (i.p.) for four consecutive days to induce loxP-dependent recombination. For mice aged 4–6 weeks, the dose was reduced to 75 mg/kg body weight with the same administration schedule.

### Tissue preparation for immunohistochemistry

The animals were deeply anesthetized with 1% pentobarbital sodium (60 mg/kg) via intraperitoneal injection and transcardially perfused with cold 0.01 M PBS and cold 4% paraformaldehyde (PFA). The brains were immediately separated from the skulls and postfixed in 4% PFA for 24 h at 4°C. For vibratome sectioning, brains were directly cut into coronal sections at the desired thickness using a Leica vibratome after post-fixation. For cryo-sectioning, brains were dehydrated in 30% sucrose in 0.01 M PBS at 4°C for 2–3 days, followed by immersion in fresh 30% sucrose for an additional day. Tissue sections containing the regions of interest were then cut at 35 or 40 μm thickness using a Leica CM1950 cryostat.

### Immunohistochemistry

The brain sections were rinsed three times in 0.01 M PBS for 10 min. The samples were blocked with 0.01 M PBS containing 0.3% Triton X-100 and 4% normal donkey serum (NDS, Jackson ImmunoResearch, Cat#: 017-000-121) at room temperature (RT) for 2 h. The samples were subsequently incubated with primary antibodies in 0.3% PBST containing 1% NDS at RT overnight. The samples were rinsed three times with 0.03% PBST for 10 min and then incubated with fluorescent dye-conjugated secondary antibodies in 0.03% PBST containing 1% NDS at RT for 2 h, together with 1:500 4',6-diamidino-2-phenylindole (DAPI, Sigma‒Aldrich, Cat#: D9542-10MG). Finally, the samples were rinsed with 0.03% PBST for 10 min three times, and the slides were mounted with antifade mounting medium. The edges of the cover glass were then sealed with nail enamel.

In the present study, the following primary antibodies were used, including rabbit anti-Iba1 (Wako, Cat#: 019-19741, 1:500), goat anti-IBA1 (Abcam, Cat#: ab5076, 1:500), goat anti-GFP (Abcam, Cat#: AB6673, 1:500), goat anti-mCherry (Biorbyt, Cat#: orb11618, 1:500), rabbit anti-GFP (Invitrogen, Cat#: A-11122, 1:1000), mouse anti-NEUN (Abcam, Cat#: ab104224, 1:500), rabbit anti-NeuroD1 (Abcam, Cat#: ab109224, 1:500), rabbit anti-RFP (Abcam, Cat#: ab62341, 1:1000), rabbit anti-DCX (Abcam, Cat#: ab18723, 1:200), mouse anti-MAP2 (Sigma, Cat#: M9942, 1:2000), and rabbit anti-TUJ1 (Sigma, Cat#: T3952, 1:2000), mouse anti-LC3B (Cell Signaling Technology, Cat#: 83506T, 1:200), and rabbit anti-p62 (Cell Signaling Technology, Cat#: 23214T, 1:500). The secondary antibodies included AF488 donkey anti-goat IgG (Jackson ImmunoResearch, Cat#: 705-545-003, 1:2000), AF488 donkey anti-rabbit IgG (Jackson ImmunoResearch, Cat#: 711-545-152, 1:2000), AF568 donkey anti-rabbit IgG (Invitrogen by Thermo Fisher Scientific, Cat#: A10042, 1:2000), AF568 donkey anti-goat IgG (Invitrogen by Thermo Fisher Scientific, Cat#: A11057, 1:2000), AF647 donkey anti-rabbit IgG (Jackson ImmunoResearch, Cat#: 711-605-152, 1:2000), AF647 donkey anti-mouse IgG (Jackson ImmunoResearch, Cat#: 715-605-151, 1:2000), and AF647 donkey anti-goat IgG (Jackson ImmunoResearch, Cat#: 705-605-003, 1:2000).

### In situ apoptosis assay

A commercial TUNEL Cell Apoptosis Detection Kit (Service bio, Cat#: G1502-50T; Elab science, Cat#: E-CK-A321) was used to detect in situ cell apoptosis. Briefly, brain sections were treated with Proteinase K (20 μg/ml) for 10 min at RT. Then, brain sections were incubated with equilibration buffer for 10 min at RT. Next, apoptotic cells were labeled by TMR-5-dUTP or FITC-dUTP labeling mix at RT and counterstained with DAPI. Positive controls were treated with DNase I, and negative controls were labeled by buffer without recombinant TdT enzyme.

### Confocal microscopy

Confocal images of the fluorescent samples were acquired via ×20 (NA 0.75), ×40 (NA 1.30), or ×60 (NA 1.40) objectives of a Nikon A2 confocal microscope. The Z-stacked focal planes were taken and maximally projected by ImageJ, whereas brightness and contrast were adjusted in ImageJ if necessary.

Tile scans of whole coronal brain sections were captured with an Olympus VS120 or VS200 system and exported from OlyVIA Software in TIFF format. Further data analysis was carried out in ImageJ.

### Two-photon surgery and imaging

Two-photon cranial window surgery was performed on mice anesthetized with isoflurane (3% for induction, 1.2–1.5% for maintenance). A 3.5-mm-diameter circular cranial window was created at the skull above the sensorimotor cortex, and a same-diameter circular D263T glass slice was implanted. The cranial window and a head bar were bonded using glue and dental cement to maintain stability. In addition to performing routine postoperative care, the mice were injected intraperitoneally with antibiotics and anti-inflammatory drugs (dexamethasone sodium phosphate injection and ceftiofur sodium for injection) for five consecutive days.

Two-photon imaging was performed on days 18, 22, 26, and 30 post-tamoxifen induction (as shown in the timeline in Figure 3A). During imaging, the mice were anesthetized with isoflurane (3% for induction, 1.2–1.5% for maintenance), and body temperature was maintained by a heating pad. Imaging was performed via a self-developed two-photon microscopy system (developed by the Institute for Translational Brain Research, Fudan University) with one resonant and one galvo scan mirror. The excitation was delivered with a Coherent Chameleon Ti-Sapphire pulsed laser. The fluorescence emission was filtered with a Semrock FF01-520/35-25, FF01-593/46 filter and detected with a Hamamatsu photomultiplier tube (H7422-40P). The EGFP signal in Cx3cr1-CreER::LSL-Neurod1-EGFP mice was excited by a 920 nm laser and imaged via a 16×, 0.8 NA water-dipping objective (Nikon). Images were acquired at a depth of approximately 100 μm below the dura using ScanImage (Pologruto et al., 2003) at step intervals of 2 μm. Each Z-axis plane image (512×512 pixels) was imaged at a consistent zoom factor (∼300×300 μm) and acquired at a 1.06 Hz frame rate.

Image processing was performed via ImageJ, and the Z-stack image containing the target cells was selected. Brightness and contrast were adjusted if needed, and the maximal projection was performed. Video processing was performed via Adobe Premiere software.

### AAV vectors, lentivirus preparation, and stereotaxic injection

The AAV vectors were purchased from Obio Technology, Ltd., and PackGene Biotech, Inc, and were as follows: *AAV1/2-CMV-EGFP, AAV5/2-CMV-EGFP*, *AAV6/2-CMV-EGFP*, *AAV8/2-CMV-EGFP*, *AAV9/2-CMV-EGFP*, *AAV2-DJ-CMV-EGFP*, *AAV2-retro-CMV-EGFP*, *AAV-PHP.eB-CMV-EGFP*, *AAV6-CX3CR1-GFP*, *AAV8-CX3CR1-GFP*, *AAV9-CX3CR1-GFP*, *AAV6-CD68-GFP*, *AAV8-CD68-GFP,* and *AAV9-CD68-GFP*.

All lentiviruses were packaged by Obio Technology, Ltd., according to the author’s design and requirements. All lentiviruses were packaged in HEK293 cells, and the packaged plasmids consisted of pMDL, VSV-G, and pREV. The following lentiviral vectors were used: *pLenti-CAG-flag-NeuroD*1, *pLenti-CAG-flag-MCS*, *pLenti-CMV-GFP, pLenti-CX3CR1-GFP,* and *pLenti-CD68-GFP*.

For intracranial injection, the mouse was anesthetized with isoflurane. The stereotaxic injection coordinates were +1.0 mm anterior/posterior, ±1.7 mm medial/lateral, and –0.75 mm dorsal/ventral.

### Traumatic brain injury

TBI was induced using the controlled cortical impact (CCI) model as previously described (Cai et al., 2025). Briefly, mice were anesthetized with ketamine (100 mg/kg) and xylazine (10 mg/kg) and placed in a stereotaxic frame (Stoelting, Wood Dale, IL, USA). Body temperature was maintained at 37.0 ± 0.5°C using a heating pad throughout the procedure. After scalp disinfection, a midline incision (1.5–2 cm) was made to expose the skull. A 4 mm craniotomy was performed over the right motor cortex, approximately 1 mm lateral to the midline, using a portable drill, with care taken to preserve the dura mater. Mice with damaged dura were excluded from further analysis. Cortical impact was delivered using a 3 mm rounded steel impactor (PinPoint PCI3000, Hatteras Instruments Inc, USA) at a velocity of 1.5 m/s, impact depth of 1.5 mm, and dwell time of 100 ms. After impact, the bone flap was sealed with sterile bone wax, and the scalp was sutured. Sham-operated mice underwent the same surgical procedures without cortical impact. Following surgery, mice were placed in a 37°C recovery chamber until fully ambulatory.

### Isolation of brain CD11b^+^ cells by magnetic-activated cell sorting (MACS)

Briefly, mice were deeply anesthetized with isoflurane and transcardially perfused with cold normal saline. Brains were harvested, rinsed briefly in cold normal saline, and mechanically minced using a blade in a brain mold. The minced tissue was transferred into a C-tube containing 3 ml of digestion buffer per brain. The digestion buffer consisted of DMEM (Gibco, REF: C11995500BT) supplemented with papain (8 U/ml, Worthington, Cat#: LS00312), DNase I (125 U/ml, Sangon, Cat#: B100649-0040), and trypsin inhibitor (1.5 mg/ml, Sigma, Cat#: T9253 or Aladdin, Cat#: A274384). Tissue dissociation was performed using a gentleMACS Octo Dissociator with Heaters (Miltenyi, 37C_ABCK1 program). The resulting cell suspension was gently triturated, and the C-tube was rinsed with 0.5% BSA in DMEM. The suspension was passed through a 70 μm cell strainer (Falcon, Cat#: CLS431751) and collected in a 50 ml tube. Cells were centrifuged at 300×*g* for 5 min at 4°C, and the pellet was resuspended in 4 ml of 30% Percoll (Cytiva, Cat#: 17544502) prepared in 0.5% BSA in DMEM. Following centrifugation at 300×*g* for 5 min at 4°C, the supernatant was carefully removed, and the cell pellet was washed once with 500 μl of 0.5% BSA in DPBS by centrifugation at 300×*g* for 5 min at 4°C. The cell pellet was then resuspended in 90 μl of 0.5% BSA in DPBS and incubated with 10 μl of anti-CD11b magnetic beads (Miltenyi, Cat#: 130-097-142) for 30 min at 4°C. The labeled cells were loaded onto an LS Column (Miltenyi, Cat#: 130-042-401) pre-equilibrated with 10 ml of 0.5% BSA in DPBS and washed with an additional 10 ml of the same buffer. The column was subsequently removed from the magnetic separator, and the magnetically labeled CD11b^+^ cells were eluted with 5 ml of 0.5% BSA in DPBS by firmly applying the plunger twice. The eluted cells were collected and centrifuged at 300×*g* for 5 min at 4°C, followed by resuspension in the appropriate buffer for cell counting and downstream experiments.

### Rotarod test

All mice were acclimated to the behavioral room for half an hour before the test. Briefly, in the training phase, the mice were placed on a rotating rod at 5 revolutions per minute (RPM) to learn how to walk forward and maintain balance. Each mouse was placed on the rod for 60 s and then returned to its home cage. This procedure was repeated three times with 10 min intervals. In the testing phase, mice were placed in separate lanes on the rod, which was set to accelerate from 4 to 40 RPM over 300 s. This test was repeated three times, with approximately 30 min intervals between trials. The latency to fall was recorded and used for subsequent statistical analysis.

### Grip strength test

All mice were acclimated to the behavioral room for half an hour before the test. In brief, we gently held the mouse by the tail and placed it with all four paws on the grip strength grid. The mouse was then gently pulled back until it released the grasp, and the holding force was recorded. This measurement was repeated six times per mouse, and the maximum force value was used for subsequent statistical analysis.

### Single-cell preparation and fluorescence-activated cell sorting (FACS)

For scRNA-seq, FACS was used to harvest GFP^+^ or tdTomato^+^ cells from tamoxifen-treated Cx3cr1-CreER::LSL-NeuroD1-GFP and Cx3cr1-CreER::Ai14 mice. Briefly, brains were dissected and cut into 1 mm³ pieces using a mouse stainless steel brain matrix (RWD). The tissue pieces were digested in 3 ml of DMEM containing 8 U/ml papain (Worthington, Cat#: LS00312), 125 U/ml DNase I (Sangon Biotech, Cat#: B100649-0040), and 1.5 mg/ml trypsin inhibitor (Sigma, Cat#: T9253 or Aladdin, Cat#: A274384) via a gentleMACS Octo Dissociator (Miltenyi) with the 37C_ABDK program.

After 30 min of digestion, the mixture was gently pipetted up and down and then filtered through a 70 µm cell strainer. The dissociated cells were centrifuged at 370×*g* at RT for 10 min, and the supernatant was discarded. The cell pellets were resuspended in 4 ml of 30% Percoll (Cytiva, Cat#: 17089101) and centrifuged again at 370×*g* and 17°C for 10 min to remove debris. After centrifugation, the supernatant was discarded, and the cells were washed once with DPBS containing 0.5% BSA. Before the cells were loaded onto the SONY MA900, they were stained with 7-AAD (BD Pharmingen, Cat#: 559925, 1:700) to label dead cells. Finally, the tdTomato^+^ 7-AAD^-^ or GFP^+^ 7-AAD^-^ cells were collected for scRNA-seq immediately.

### scRNA-seq data analysis

scRNA-seq was conducted via the MobiCube High-throughput Single Cell 3′ Transcriptome Set V2.1 (PN-S050200301) and the MobiNova-100 microfluidic platform. The single-cell suspension was adjusted to a concentration of 700–1200 cells/μl and immediately loaded onto a chip for microdroplet formation via the MobiNova-100 system. Reverse transcription, cDNA amplification, and DNA library construction were carried out according to the manufacturer’s protocol. High-throughput sequencing was performed in PE-150 mode.

FASTQ files were processed and aligned to the mouse reference genome (mm10) via MobiVision version 3.2, with unique molecular identifier (UMI) counts aggregated for each barcode. The resulting UMI count matrix was analyzed in R version 4.2.2 via Seurat version 4.3.0. To ensure data quality, we excluded low-quality cells and potential multiplets on the basis of the following criteria: cells with fewer than 200 genes or more than 5000 genes and cells with more than 10% UMIs mapped to mitochondrial genes. scRNA-seq data from the four groups were integrated via *IntegratedData* functions according to the manufacturer’s instructions. The remaining data were normalized via the *NormalizeData* function and scaled with *ScaleData*. For scRNA-seq analysis, principal component analysis (PCA) was conducted on variable features, with the top 30 principal components (PCs) selected for clustering and visualization via tSNE and uniform manifold approximation and projection (UMAP). Cell clustering was performed with a resolution of 1 to annotate the cell types. Differentially expressed genes (DEGs) among cell groups were identified via the *FindMarkers* function with parameters set to min.pct=0.1 and a log fold change (FC) threshold of 0.25 (Wilcoxon rank-sum test). Genes with |log2FC|>0.25 and p<0.05 were considered significant DEGs and subsequently used for Gene Ontology (GO) analysis. Visualization of the results was performed via the *VlnPlot* and *FeaturePlot* functions. We gratefully thank OE Biotech Co., Ltd. (Shanghai, China) for providing sequencing service.

### Statistical analysis

Statistical analyses were performed with Prism 10.2.0 (GraphPad). In histological quantification, each data point represents the average statistical result of more than three brain sections in different bregma regions of one mouse. The results were evaluated independently in a double-blind manner. All the results are presented as the means ± standard deviations (SDs). One-way analysis of variance (ANOVA) with Tukey’s multiple comparisons test was performed for multiple comparisons, whereas a two-tailed independent *t*-test was used to compare the differences between two groups. For the behavior experiment, two-way ANOVA with multiple comparisons was used. No outliers were excluded.

### Primer information

**Table:** 

Gene	Sequence (5′→3′)
*Aif1*	Forward: GACTGCCAGCCTAAGACA
	Reverse: AGGAATTGCTTGTTGATCCC
*P2ry12*	Forward: ATGGATATGCCTGGTGTCAACA
	Reverse: AGCAATGGGAAGAGAACCTGG
*Neurod1*	Forward: TTCAAACACGAACCATCCGC
	Reverse: GTCTATGGGGATCTCGCAGC
*Neurod1*	Forward: ATAGAGACACTCGCCTTGGC
	Reverse: GCGTCTGTACGAAGGAGACC
*Neurod1*	Forward: AAGCCACGGATCAATCTTCTC
	Reverse: GAATAGTGAAACTGACGTGCCT
*Atg5*	Forward: AGCCAGGTGATGATTCACGG
	Reverse: GCCTGGGGGACAATGCTAA
*Atg5*	Forward: TGTGCTTCGAGATGTGTGGTT
	Reverse: GTCAAATAGCTGACTCTTGGCAA
*Gapdh*	Forward: AGGTCGGTGTGAACGGATTTG
	Reverse: TGTAGACCATGTAGTTGAGGTCA

### Declaration of generative AI and AI-assisted technologies in the manuscript preparation process

As the authors are non-native English speakers, the authors used ChatGPT 4.0, 5.5, and WorkBuddy 5.5.6 during the preparation of this manuscript in order to improve the language and enhance its readability. After using this tool, the authors reviewed and edited the content as needed and take full responsibility for the content of the published article.

## Data Availability

The sequencing data generated in this study have been deposited in the Gene Expression Omnibus under accession number GSE277222. The following dataset was generated: LiX RaoY PengB 2026scRNA-seq data from normal microglia and NeuroD1-overexpressing microglia under both normal and TBI conditionsNCBI Gene Expression OmnibusGSE277222

## Additional files

MDAR checklist
