# Supplementary material for: In vivo virus-free lineage tracing and live imaging reveal that NeuroD1 does not reprogram microglia into neurons
Source: eLife. 2026 Oct 16;15:RP112083. doi: 10.7554/eLife.112083 (PMC13675009; doi:10.7554/eLife.112083)
Supplement: Peer review history

# Editor report: eLife Assessment

**DOI:** 10.7554/eLife.112083.3.sa0

**Authors:** Florent Ginhoux

**Affiliations:** Singapore Immunology Network Singapore

**Keywords:** Compelling, Important

This is an **important** study addressing a debated question in brain repair research by testing whether NeuroD1 can convert brain immune cells into nerve cells using a virus-free genetic approach and live imaging. The **compelling** evidence presented here supports that, under the conditions tested, NeuroD1-expressing cells do not become nerve cells and instead retain their original identity. This work will be of interest to neuro-immunologists.

# Referee report: Reviewer #1 (Public review)

**DOI:** 10.7554/eLife.112083.3.sa1

Summary:

This study revisits an important and controversial question in brain repair: whether NeuroD1 can convert brain immune cells into nerve cells in vivo. Using a virus-free genetic system, in vivo imaging, injury experiments, and single-cell profiling, the authors provide convincing evidence that NeuroD1-expressing cells do not become nerve cells under the tested conditions. Instead, these cells largely retain their original immune-cell identity, and some appear to undergo cellular stress or loss.

Strengths:

The main strength of the work is that it tests this question with a cleaner genetic strategy, avoiding some of the concerns associated with viral delivery and unintended cell labeling. Although the overall conclusion is consistent with the authors' previous work, the current study adds useful independent evidence, particularly through the virus-free fate-mapping system and live imaging in the brain.

Weaknesses:

The tested time window cannot fully exclude the possibility of very delayed or incomplete neuronal differentiation.

Overall, this is a useful and careful study that supports the conclusion that NeuroD1 does not drive brain immune cells to become nerve cells in the tested settings. It should be valuable for researchers studying brain repair, cell fate conversion, and genetic fate mapping, and it provides a clear caution against overinterpreting reprogramming results based only on viral labeling.

# Referee report: Reviewer #2 (Public review)

**DOI:** 10.7554/eLife.112083.3.sa2

Summary:

In vivo glia-to-neuron conversion emerges as a potential regeneration-based therapeutic strategy for neural injuries and diseases. However, controversies exist in this exciting field, largely arising from the non-stringent methods use to analyze in vivo neuronal conversions. The study by Li et al. directly tackled such a controversy on Neurod1-mediated microglia-to-neuron conversion. They took advantage of transgenic mouse lines to specifically express Neurod1 in microglia of adult mouse brains. Results from immunohistology, in vivo live cell imaging, and scRNA-seq convincingly demonstrate that microglia cannot be converted in vivo to neurons by ectopic Neurod1 expression under the specified normal or injury conditions. Instead, it induces microglia death, consistent with their earlier findings. These solid results, though negative, are critical additions to the research field and further support that stringent lineage tracing methods are essential for studying in vivo cell reprogramming. Overall, the studies are rigorously designed and executed.

# Author comment: Author response

**DOI:** 10.7554/eLife.112083.3.sa3

**Authors:** Xiaoyu Li, Yuxin Li, Yue Cao, Niewen Hu, Baozhi Yang, Pei Ouyang, Yuxiao Jin, Shuai Gao, Li Cao, Bo Peng, Yanxia Rao

**Affiliations:** Fudan University Shanghai China; Fudan University Shanghai China; Fudan University Shanghai China; Fudan University Shanghai China; Fudan University Shanghai China; Fudan University Shanghai China; Fudan University Shanghai China; Fudan University Shanghai China; Navy Military Medical University Shanghai China; Fudan University Shanghai China; Fudan University Shanghai China

The following is the authors’ response to the original reviews.

**Public Reviews:**

**Reviewer #1 (Public review):**

Summary:

This study revisits an important and controversial question in brain repair: whether NeuroD1 can convert brain immune cells into nerve cells in vivo. Using a virus-free genetic system, in vivo imaging, injury experiments, and single-cell profiling, the authors provide convincing evidence that NeuroD1-expressing cells do not become nerve cells under the tested conditions. Instead, these cells largely retain their original immune-cell identity, and some appear to undergo cellular stress or loss.

Strengths:

The main strength of the work is that it tests this question with a cleaner genetic strategy, avoiding some of the concerns associated with viral delivery and unintended cell labeling. Although the overall conclusion is consistent with the authors' previous work, the current study adds useful independent evidence, particularly through the virus-free fate-mapping system and live imaging in the brain.

Weaknesses:

There are some limitations. In the injury experiment, the labeled cells may include both resident brain immune cells and blood-derived immune cells recruited after injury, so the authors should be cautious when referring to all labeled cells as microglia. The level of NeuroD1 expression achieved by the genetic system is also not fully defined, which matters because the effects of such a cell-fate regulator may depend on expression level. Finally, the tested time window may not fully address very delayed or incomplete neuronal differentiation.

Overall, this is a useful and careful study that supports the conclusion that NeuroD1 does not drive brain immune cells to become nerve cells in the tested settings. It should be valuable for researchers studying brain repair, cell fate conversion, and genetic fate mapping, and it provides a clear caution against overinterpreting reprogramming results based only on viral labeling.

We’d like to thank the reviewer for providing these thoughtful suggestions and positive assessment of our study. We agree that CX3CR1 lineage can label both microglia and blood-derived immune cells after injury, To more specifically assess microglial lineage tracing and minimize the potential contribution of infiltrating peripheral myeloid cells, we performed additional experiments using TMEM119-CreER::LSL-NeuroD1-EGFP mice. These experiments showed no evidence of microglia-to-neuron conversion following TBI. To evaluate the expression level of NeuroD1, we performed qPCR to verify the NeuroD1 expression level in the CD11b^+^ cell population of the NeuroD1-expressing mice, and found that NeuroD1 expression was increased approximately 6.21-fold compared with control mice, indicating robust NeuroD1 expression. For the question about the time window, we have revised our Discussion and Conclusion parts to avoid overgeneralizing our findings beyond the experimental conditions tested. We agree that our data cannot fully exclude the possibility of delayed neuronal differentiation or neuronal conversion under other pathological conditions. Besides, we have also revised the manuscript accordingly to address the concerns raised by the reviewer.

**Reviewer #2 (Public review):**

Summary:

In vivo glia-to-neuron conversion emerges as a potential regeneration-based therapeutic strategy for neural injuries and diseases. However, controversies exist in this exciting field, largely arising from the non-stringent methods employed for analyzing in vivo neuronal conversions. The study by Li et al. directly addressed this controversy regarding Neurod1-mediated microglia-to-neuron conversion. They took advantage of two transgenic mouse lines to specifically express Neurod1 in the microglia of adult mouse brains. Results from immunohistochemistry, in vivo live-cell imaging, and scRNAseq convincingly demonstrate that microglia cannot be converted in vivo to neurons by ectopic Neurod1 expression under both normal and injury conditions. Instead, it induces microglia death, consistent with their earlier findings. These solid results, though negative, are critical additions to the field and further support that stringent lineage tracing methods are essential for studying in vivo cell reprogramming. Overall, the studies are rigorously designed and executed. Only minor issues need to be dealt with.

We’d like to thank the reviewer for providing these thoughtful comments and for the positive assessment of our study. We are glad that the reviewer agrees that our results from immunohistochemistry, in vivo live-cell imaging, and scRNA-seq support the conclusion that microglia cannot be converted into neurons by NeuroD1 expression under the tested conditions. We have carefully addressed the minor issues raised by the reviewer, including rechecking the grammar throughout the manuscript, revising the description of the TBI behavioral results, discussing the limitations of scRNA-seq for neuronal detection, adding the information of the promoters used in the study, and carefully revising the references. Besides, we have carefully revised the manuscript according to the reviewer’s suggestions.

**Recommendations for the authors:**

**Reviewer #1 (Recommendations for the authors):**

Major comments

(1) TBI lineage tracing may label both microglia and infiltrating macrophages. Because tamoxifen was administered after TBI, CX3CR1-CreER may label not only resident microglia but also injuryrecruited CX3CR1+ monocytes/macrophages. The authors should avoid referring to all reporterpositive cells as microglia unless this is clearly supported. They should clarify the composition of reporter-positive cells in the TBI setting, ideally using existing scRNA-seq data or markers distinguishing microglia from infiltrating macrophages.

The current data still support the conclusion that CX3CR1-lineage myeloid cells do not show obvious neuronal conversion in this TBI model, but the wording should be more precise.

Thank you for pointing this out. We agree that, because TBI can recruit CX3CR1-expressing monocytes/macrophages, CX3CR1-CreER lineage tracing after injury may label both resident microglia and infiltrating myeloid cells. To more specifically assess the contribution of resident microglia, we therefore performed additional lineage-tracing experiments using TMEM119CreER::Ai14 (TMEM119-Ai14) and TMEM119-CreER::LSL-NeuroD1-EGFP(TMEM119-ND1) mice, with tamoxifen administration before or after TBI. In both experimental settings, we did not detect reporter-positive cells co-expressing the neuronal marker NeuN in either the lesion core or distal regions. These results provide no evidence of microglia-to-neuron conversion in the TBI model within the examined time window. We have also revised the text throughout the manuscript to distinguish resident microglia from broader CX3CR1-lineage myeloid cells where appropriate.

**Author response image 1.** TMEM119-CreER::LSL-NeuroD1-EGFP animal also show no microglia-to-neuron conversion in TBI. (A) Tamoxifen induction after TBI shows there is no GFP^+^NeuN^+^ cells in both lesion core and distal region. (B) Tamoxifen induction before TBI shows there is no GFP^+^NeuN^+^ cells in both lesion core and distal region.

(2) NeuroD1 expression level should be better characterized. The authors show that GFP-positive cells co-express NeuroD1, but the approximate NeuroD1 expression level is not clear. This is important because the effect of a fate-determining transcription factor may be dose-dependent. The authors should provide, if possible, a quantitative estimate of NeuroD1 expression using available IF, scRNA-seq, qPCR, or other data. This would help interpret the negative reprogramming result and would also be useful for future studies using this Rosa26-LSL-NeuroD1-IRES-GFP mouse line.

Thank you for this valuable suggestion. To quantitatively assess NeuroD1 expression in the myeloid compartment, we isolated CD11b^+^ cells from CX3CR1-CreER::Ai14 (CX3CR1-Ai14) and CX3CR1-CreER::LSL-NeuroD1-EGFP (CX3CR1-ND1) mice using MACS and performed qPCR analysis of *Neurod1* expression. *Neurod1* expression was increased approximately 6.21-fold in CD11b^+^ cells from CX3CR1-ND1 mice compared with the corresponding control mice (Author response image 2). These results confirm robust NeuroD1 expression in the CD11b^+^ cell population of the NeuroD1-expressing mice. This level of induction is broadly comparable to the approximately 4-fold increase in *Neurod1* expression reported following NeuroD1 induction in the intestine, in which the same LSL-NeuroD1-EGFP transgenic mouse line was used [1].

**Author response image 2.** Using qPCR verify the relative expression of *Neurod1*.

(3) Figure 4 scRNA-seq should show Neurod1 expression. The scRNA-seq data show that reporterpositive cells retain microglial markers and lack neuronal markers, but Neurod1 expression itself is not shown. The authors should consider adding Neurod1 feature plots, violin plots, or average expression comparisons between control and NeuroD1-expressing groups. This would help validate the expression system and estimate the level achieved in sorted reporter-positive cells.

Thank you for your suggestion. We examined the expression of *Neurod1* in our scRNA-seq database, but the *Neurod1* transcripts were rarely detected (not just in this database, also our previous dataset). This might because of *Neurod1* is a transcription factor and single-cell RNA-seq method is not suitable for detect these low-expression transcript factor genes, so we utilized qPCR to evaluate the expression of *Neurod1*, as shown in Author response image 2.

(4) The microglial loss phenotype may be related to NeuroD1/EGFP dosage. In Figure 3, the loss of GFP-positive microglia may reflect a specific effect of NeuroD1 in microglia, but it could also be related to excessive transgene expression or expression-system toxicity. The authors should discuss this possibility and, if possible, examine whether stress/death markers correlate with NeuroD1 or GFP expression intensity.

Thanks for your suggestion. We agree with that, so we performed TUNEL staining on CX3CR1-CreER::Ai14 (CX3CR1-Ai14 for short) and CX3CR1-CreER::LSL-NeuroD1EGFP(CX3CR1-ND1 for short) mice at D4 and D18. The data shows that TUNEL^+^ Reporter^+^ cells were higher in NeuroD1-expressing microglia, so we’d like to say death cells (or apoptotic cells) are positively correlated with NeuroD1 expression. Besides, we also discussed more about this. See Figure 3 E, F, G and Fig. S3E

(5) The time window and injury context should be more cautiously discussed. The study follows the genetic model up to 45 days. This is informative, but delayed or incomplete neuronal differentiation cannot be fully excluded, especially if conversion would require a longer repair phase after injury. In addition, except for TBI, the study does not include another neuronal-loss context that might provide a permissive regenerative niche. The authors do not necessarily need additional long-term experiments, but they should avoid overgeneralizing beyond the tested time window and injury model.

Thank you for this thoughtful comment. We agree that our study evaluated the effects of NeuroD1 expression within a defined experimental time window (up to 45 days) and under the specific physiological and injury conditions examined. Therefore, our data cannot exclude the possibility that neuronal conversion might occur at later time points or under other pathological conditions that provide a more permissive regenerative environment. In response to the reviewer's suggestion, we have revised the Discussion and Conclusion to avoid overgeneralizing our findings beyond the experimental conditions tested. We now emphasize that NeuroD1 expression alone did not induce microglia-to-neuron conversion within the time frame and injury paradigms examined in this study, rather than concluding that such conversion can never occur under other conditions.

Minor comments

(1) In Figure 2, two panels are labeled "D"; the second should likely be "E."

Thank you for pointing this out, we have corrected it in figure legend.

(2) Several spelling and grammar errors should be corrected, such as "Represent images," "illutrating," and "GFP-postive."

Thank you for pointing this out, we have corrected it in our manuscript.

(3) More scRNA-seq details should be provided, including cell numbers per group, QC metrics, and cluster annotation information.

Thanks for your suggestion, we have added more details about scRNA-seq data as shown in Figure S4.

(4) The authors should maintain cautious wording throughout, especially when referring to "microglia" versus broader CX3CR1-lineage myeloid cells.

Thanks for your suggestion, we added the data about we utilize TMEM119-ND1 mouse line to trace microglia-to-neuron conversion, which is specific to microglia not myeloid cell. Besides, we also carefully revise these minor comments in our manuscript.

**Reviewer #2 (Recommendations for the authors):**

The following are some required minor edits.

(1) Please recheck the grammar throughout the manuscript.

Thank you for pointing this out, we have carefully revised our manuscript.

(2) Figure 4B-C: "indicating that NeuroD1 expression in microglia does not rescue TBI induced motor dysfunction". This should be rephrased, since dysfunction was not detected when comparing TBI and sham.

Thank you for pointing this out, we changed our expression into “indicating that NeuroD1 expression in microglia does not improve the performance in TBI model”.

(3) Interpretation of the scRNA-seq data needs to be cautious, since neurons normally do not survive well during this procedure.

Thank you for your suggestion, we discussed about this in our discussion part.

(4) It will be informative to list the sources or sequences of the promoters used.

Thanks for your advice, all the virus information are provided in our method part.

(5) Some of the references are repetitive.

Thank you for your suggestion, we have carefully revised the reference.

Reference

(1) Li, H. J. et al. Intestinal Neurod1 expression impairs paneth cell differentiation and promotes enteroendocrine lineage specification. Sci Rep **9**, 19489 (2019). https://doi.org/10.1038/s41598-019-55292-7
